# The Interaction of Src Kinase with β3 Integrin Tails: A Potential Therapeutic Target in Thrombosis and Cancer

**DOI:** 10.1100/tsw.2010.114

**Published:** 2010-06-15

**Authors:** Stephan Huveneers, Erik H. J. Danen

**Affiliations:** ^1^ Dynamics of Cell Adhesion, Hubrecht Institute, University Medical Centre Utrecht, The Netherlands; ^2^ Division of Toxicology, Leiden Amsterdam Center for Drug Research, Leiden University, The Netherlands

**Keywords:** β3 integrin, c-Src, platelet, cancer, therapy

## Abstract

Activation of Src family kinases is an important event downstream of integrin adhesion signaling in many cell types. A particularly intriguing connection between an integrin and a Src family kinase was first discovered in platelets, where the selective direct interaction of αIIbβ3 integrins with c-Src promotes full kinase activation of c-Src through its local clustering by the cytoplasmic tail of the β3 integrin subunit. The same integrin β3-c-Src interaction not only drives platelet aggregation, but it also promotes the oncogenic potential of c-Src and drives tumor growth by αvβ3-expressing tumor cells, which may explain why increased activity of c-Src and elevated levels of integrin αvβ3 are often found in the same tumor types. Moreover, recent evidence from patient material and in vivo studies strongly indicate that this oncogenic signaling complex, consisting of c-Src and αvβ3, underlies tumor progression of human tumors. Here, we give an overview of the β3-c-Src interaction and its implications for signaling in platelets and tumor cells, and we mention the possibilities for therapeutic intervention that is aimed at disrupting the β3-c-Src interaction for antithrombotic and anticancer purposes.

## INTRODUCTION

Cells interact with the extracellular matrix through various adhesion receptors, including integrins. Integrins are heterodimeric transmembrane adhesion receptors that link the extracellular matrix with intracellular signaling molecules. When binding to their ligands, integrins cluster and organize the formation of multiprotein complexes, at sites of adhesion, which propagate signaling cascades towards a range of crucial cellular processes involved in normal tissue function and disease[1,2].

Members of the Src family of tyrosine kinases (SFKs) localize to adhesion complexes where they regulate protein-protein interactions and thereby control adhesion turnover. SFKs also control signaling pathways downstream of integrins involved in cytoskeletal organization[3]. Most SFKs are targeted to integrin adhesion complexes indirectly; for instance, through binding to focal adhesion kinase (FAK) or growth factor receptors. c-Src has also been reported to bind directly to the β-cytoplasmic tails of αIIbβ3

and αvβ3 integrins, which contributes to unfolding and activation of c-Src. Here we describe the cellular functions of this β3-c-Src signaling unit, and discuss its importance for platelet function as well as growth and progression of cancer.

## INTEGRIN αIIbβ3 SPECIFICALLY ACTIVATES c-SRC TO INDUCE PLATELET SPREADING

Inactive c-Src is folded in a closed conformation due to two intramolecular interactions: (1) Csk-mediated phosphorylation of the C-terminal tyrosine 530 (pY530) creates a binding site for the SH2 domain, and (2) the proline-rich linker region between the SH2 and kinase domain binds to the membrane proximal SH3 domain. Release of both interactions opens up the molecule towards a "primed conformation" that can be cross-phosphorylated by other Src molecules on tyrosine 419 in the activation loop of the kinase domain leading to full kinase activity[4]. Functional associations between β3 integrins and c-Src have been described[5,6,7]. In platelets, engagement of integrin αIIbβ3 by its ligand fibrinogen stimulates c-Src activation, which in turn stimulates Syk-mediated cytoskeletal reorganization and platelet spreading on fibrinogen[8]. Direct binding of c-Src to the β3 cytoplasmic tail was first demonstrated biochemically for αIIbβ3 in platelets[9]. This involves a selective atypical interaction of the c-Src SH3 domain with the C-terminal YRGT residues of the β3 cytoplasmic domain[9]. This interaction competes with the c-Src proline-rich linker region for binding to the c-Src SH3 domain, thereby supporting the primed conformation of c-Src in synergy with interactions that compete with Src pY530 for binding to the Src SH2 domain; for instance, mediated by phosphorylated tyrosines in receptor tyrosine kinase (RTK) cytoplasmic tails. Binding of c-Src to the β3 tail appears to be constitutive, suggesting that enhanced priming is ligand independent, but ligand-mediated clustering of αIIbβ3 integrins drives the final activation of c-Src by promoting transphosphorylation of tyrosine 419 residues in the Src kinase domain[9]. This direct interaction between c-Src and αIIbβ3 can explain their dual requirement for platelet spreading on fibrinogen: adhesion of activated platelets to fibrinogen induces clustering of αIIbβ3 integrins, activation of c-Src, and downstream signaling through Syk, which is responsible for platelet aggregation and thrombus formation.

## INTEGRIN αvβ3 CONTROLS THE ONCOGENIC ACTIVITY OF PRIMED c-SRC TO SUPPORT TUMOR GROWTH

Increased expression and activation of c-Src is associated with poor prognosis in various cancer types[10,11]. In those same types of cancer, increased expression of integrin αvβ3 is related to tumor growth[12]. Overexpressed RTKs in tumor cells stimulate priming of c-Src by competitive binding to the Src SH2 domain, which weakens the intramolecular SH2-pY530 interaction[13]. We have found that increased expression of αvβ3 promotes wild-type c-Src–induced tumor growth in the context of overexpressed epidermal growth factor receptor[14]. We expected that this was due to synergy between RTK interactions with the Src SH2 domain and the described β3 interactions with the Src SH3 domain, which would maximize unfolding and priming of c-Src causing oncogenic signaling towards tumor growth. To address this, we investigated the oncogenic properties of a primed mutant of c-Src (SrcY530F), which mimics the primed state induced by overexpression of RTKs, and corresponds to C-terminal mutants of c-Src found in subsets of patients with colon and endometrial cancer[15,16]. By using cells expressing different types of integrins, we discovered that the activity of primed c-Src is strongly augmented upon increased expression of αvβ3, which drives anchorage-independent growth and subcutaneous tumor growth. In analogy to the αIIbβ3-c-Src interaction in platelets, the αvβ3-c-Src oncogenic interaction required the cytoplasmic terminus of β3 and the SH3 domain of c-Src. Notably, the oncogenic potential of primed c-Src could not be supported by β1 integrins and RasV12-driven tumor growth was not affected by the absence or presence of αvβ3, indicating that c-Src and αvβ3 form a unique oncogenic signaling unit[14]. Thus, integrin αvβ3 on tumor cells can promote (primed) c-Src activation and tumor growth, perhaps explaining why the expression of these two proteins is associated with poor prognosis, and implicating that interfering with their interaction might be a valuable therapeutic goal.

## INTEGRIN αvβ3 AND c-SRC DRIVE TUMOR METASTASIS AND PROGRESSION

There is a particularly strong correlation between elevated c-Src kinase activity[11,17] and increased levels of integrin αvβ3[12] with tumor progression. Increased c-Src activity not only induces tumorigenicity, it also drives dramatic morphological changes that may contribute to tumor progression. We found that primed c-Src equally stimulates the formation of highly dynamic invasion structures, called podosomes, both in cells expressing αvβ3 or α5β1, although podosome distribution is different[18]. In addition, integrin αvβ3 protects against Src-mediated inhibition of cell spreading. The influence of integrins on the morphological alterations induced by primed c-Src may contribute to tumor invasiveness.

It was recently demonstrated that the oncogenic signaling complex of integrin αvβ3 and c-Src promotes tumor progression of human pancreatic and breast carcinomas. Expression of integrin αvβ3 was enriched in human lymph node metastasis compared to the matched primary pancreatic and breast tumors[19]. In accordance with our findings, increased αvβ3 expression in pancreatic tumor cells induced enhanced primary tumor growth and anchorage-independent growth, which occurred through c-Src recruitment to the β3 cytoplasmic domain. This interaction caused c-Src activation and downstream signaling through Crk-associated substrate (CAS). Intriguingly, although adhesion of the pancreatic tumor cells to the extracellular matrix protein fibronectin was mediated through both α5β1 and αvβ3 integrin types, activated Src only colocalized with αvβ3 integrins. Importantly, silencing of c-Src expression or pharmacological blockade of c-Src activity using dasatinib, but not inhibition of the related protein FAK, inhibited spontaneous metastasis of αvβ3-expressing tumor cells. Moreover, reduced expression of αvβ3, but not blockade of αvβ3 ligand binding, lowered anchorage-independent growth and metastasis[19].

There is accumulating evidence for a role of the αvβ3 and c-Src complex in tumor progression of other cancers. For instance, expression of high-affinity state integrin αvβ3 supported growth of metastatic brain tumors by a strong induction of VEGF production and tumor angiogenesis[20]. Although this still has to be investigated thoroughly, the enhanced effect on tumor metastasis by activated αvβ3 may well occur through oncogenic signaling through the β3-c-Src oncogenic complex, as activation of platelet integrin αIIbβ3 is indeed associated with increased activation of c-Src[8]. Another example comes from analysis of different melanoma cell types. Expression of integrin αvβ3 alone in melanoma cells turned out to be insufficient to support invasion of melanoma cells; instead, elevation of c-Src activity was strictly required to support αvβ3-mediated invasion[21].

Together, these studies indicate that the selective interaction of β3 and c-Src may be a very potent therapeutic target to treat tumor growth, invasion, and metastasis.

## MODEL OF SRC ACTIVATION BY β3 INTEGRINS

The following c-Src activation model by β3 integrins can be proposed (Fig. 1): c-Src in unstimulated cells is maintained in an inactive conformation through Csk-mediated phosphorylation of tyrosine 530, and through SH2- and SH3-mediated intramolecular interactions. The cytoplasmic tails of β3 integrins bind constitutively and selectively to the SH3 domain of c-Src, creating a pool of Src molecules that are partly primed. Whether this interaction promotes more extensive conformational changes and leads to the recruitment of additional proteins that contribute to c-Src activation is unclear. Other events, for instance, Csk inactivation and/or competitive SH2-mediated interactions with pY530, are required to disrupt the SH2-pY530 intramolecular interaction, thereby contributing to further priming. However, the interaction with the β3 cytoplasmic tail is critical for full c-Src activation, since activation of a SrcY530F mutant still depends on integrin αvβ3 for its activity, despite the fact that autoinhibition of pY530 with the SH2

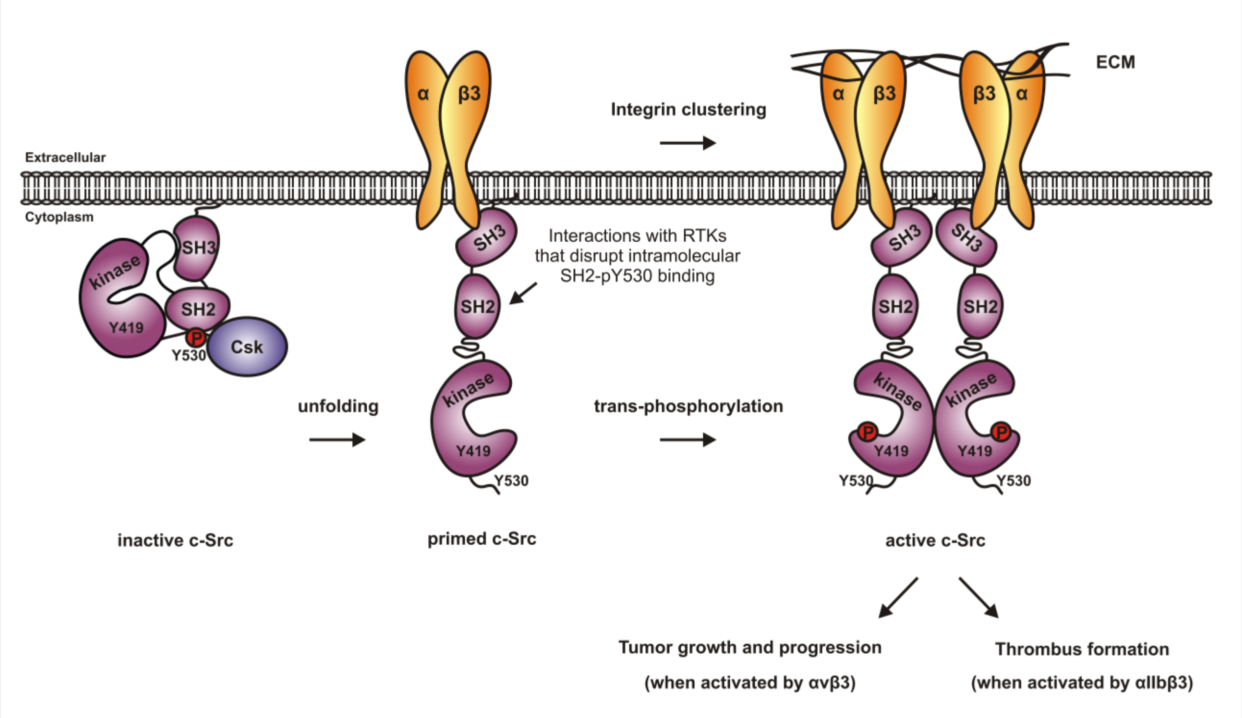

**FIGURE 1**. Model of c-Src activation by β3 integrins.

domain is abrogated. As a final step, ligand-mediated clustering of β3 integrins may promote full activation of c-Src through transphosphorylation of the c-Src kinase domain.

In platelets, activation of c-Src by αIIbβ3 requires fibrinogen-mediated clustering[22]. Our findings with c-Src in the context of overexpressed RTKs or primed SrcY530F mutants, and the results from Desgrosellier and colleagues investigating c-Src in pancreatic cancer cells, indicate that αvβ3 integrin can support the oncogenic potential of c-Src in an anchorage-independent way[14,19]. However, a role for ligand-induced activation of integrins in the activation of c-Src cannot be excluded, and ligands in solution or trapped between cells may play a critical role in these experimental setups. On the other hand, the β3 integrin subunit has been reported to have a tendency to form homo-oligomers in the plane of the plasma membrane, providing a possible ligand-independent mechanism for c-Src clustering[23].

## THE BINDING INTERFACE OF β3 AND c-SRC AS A PROMISING THERAPEUTIC TARGET

*In vitro* experiments and preclinical models show promising effects of c-Src as a therapeutic target to inhibit tumor growth and progression, and several Src inhibitors are currently being tested in clinical trials[24]. Similarly, integrin antagonists may be valuable as adjuvants to increase the efficacy of radio- and chemotherapy[25]. The discovery of the importance of the direct interaction of αvβ3 and c-Src for tumor growth and progression raises the opportunity for the development of highly specific inhibitors for tumor types in which αvβ3 levels and c-Src activity are elevated. The C-terminal RGT residues of the β3 cytoplasmic domain and the SH3 domain of c-Src might form the basis of the development of such β3-c-Src–specific inhibitors. Again, much might be learned from studies in platelets, as it was recently found that a cell-permeable and membrane-targeted RGT peptide indeed prevents the interaction of αIIbβ3 and c-Src in human platelets, thereby lowering c-Src activation and platelet spreading on fibrinogen, indicating that this peptide may be a promising antithrombotic agent[26]. In analogy, we propose that such a developed drug targeted to tumor cells may also be useful for treatment of cancer.

## ACKNOWLEDGMENTS

The work of SH and EHJD is supported by the Dutch Cancer Society (grants UL2006-3521 and UL2007-3860).
